# Applications of Artificial Intelligence to Popularize Legal Knowledge and Publicize the Impact on Adolescents' Mental Health Status

**DOI:** 10.3389/fpsyt.2022.902456

**Published:** 2022-05-26

**Authors:** Hao Liu

**Affiliations:** School of Law, Chongqing University, Chongqing, China

**Keywords:** artificial intelligence, legal knowledge, cognitive fuzzy K-nearest neighbor, variational autoencoder, fitness-based logistic regression analysis, MATLAB tool

## Abstract

Artificial intelligence (AI) advancements have radically altered human production and daily living. When it comes to AI's quick rise, it facilitates the growth of China's citizens, and at the same moment, a lack of intelligence has led to several concerns regarding regulations and laws. Current investigations regarding AI on legal knowledge do not have consistent benefits in predicting adolescents' psychological status, performance, etc. The study's primary purpose is to examine the influence of AI on the legal profession and adolescent mental health using a novel cognitive fuzzy K-nearest neighbor (CF-KNN). Initially, the legal education datasets are gathered and are standardized in the pre-processing stage through the normalization technique to retrieve the unwanted noises or outliers. When normalized data are transformed into numerical features, they can be analyzed using a variational autoencoder (VAE) approach. Multi-gradient ant colony optimization (MG-ACO) is applied to select a proper subset of the features. Tree C4.5 (T-C4.5) and fitness-based logistic regression analysis (F-LRA) techniques assess the adolescent's mental health conditions. Finally, our proposed work's performance is examined and compared with classical techniques to gain our work with the greatest effectiveness. Findings are depicted in chart formation by employing the MATLAB tool.

## Introduction

A person's mental health is defined as social well-being, psychological and emotional. It impacts how a person thinks, feels, and reacts to situations. It is simpler to perform effectively and realize one's full potential when one has good mental health. Mental well-being at any age includes preschool, adolescence, and adulthood. Anxiety, social phobia, sadness, panic disorder, drug dependency, and individual diseases are all components that contribute to mental health issues that lead to mental disease. It is becoming more important to notice the onset of mental illness to maintain a healthy life balance. An individual's mental well-being is a combination of their mental state and an assessment of their whole personality. Chemical imbalances cause mental diseases in the brain. Understanding and treating persons with abnormal mental activity requires a detailed study of their mental health (1).

Mental health serves as a gauge for how effectively they are being addressed for their illnesses. It's vital to maintain track of different groups' mental health profiles to predict health-related anomalies. High school pupils, college graduates, and young professionals make up the community. Persons and backgrounds are said to be affected by stress and unhappiness. It is vital to maintain the mental health of distinct groups at different times to prevent catastrophic illness. According to a 2011 study by the ‘World Health Organization (WHO) executive council, depression will be the leading cause of global illness burden by 2030. Healthcare providers will be obliged in the next years to consider a sufferer's mental health status to provide better treatment and help in a faster recovery. Winters-Miner and colleagues investigated how prescriptive medical modeling would impact the worldwide healthcare business (2).

Psychologists devote a lot of time to psychotherapy and behavioral interventions to help patients with emotional and mental problems. Psychologists are also competent to perform psychological testing, which is essential for establishing a person's mental condition and the most successful treatment plan. Professionals will be able to use the developed prediction technique to conduct psychological sampling and forecast a person's mental health. The psychologist and psychiatrist work together to treat the participant's behavior and clinical problems. Psychologists and psychiatrists are both important in researching and advancing mental and emotional health solutions (3).

Because all except the most severe psychiatric therapy is provided in an outpatient environment, greater community monitoring would be very beneficial. Early identification and prevention of recurrence may make a big difference in how things turn out. Patients' written records are one way to track emotion and mental health outside of the professional context. There is a risk of recollection bias and compliance concerns with this method. Although some of the same challenges exist, apps have been created that actively urge users to answer questions about their mood, sleeping habits, and other pertinent areas (4).

**Figure 1:**
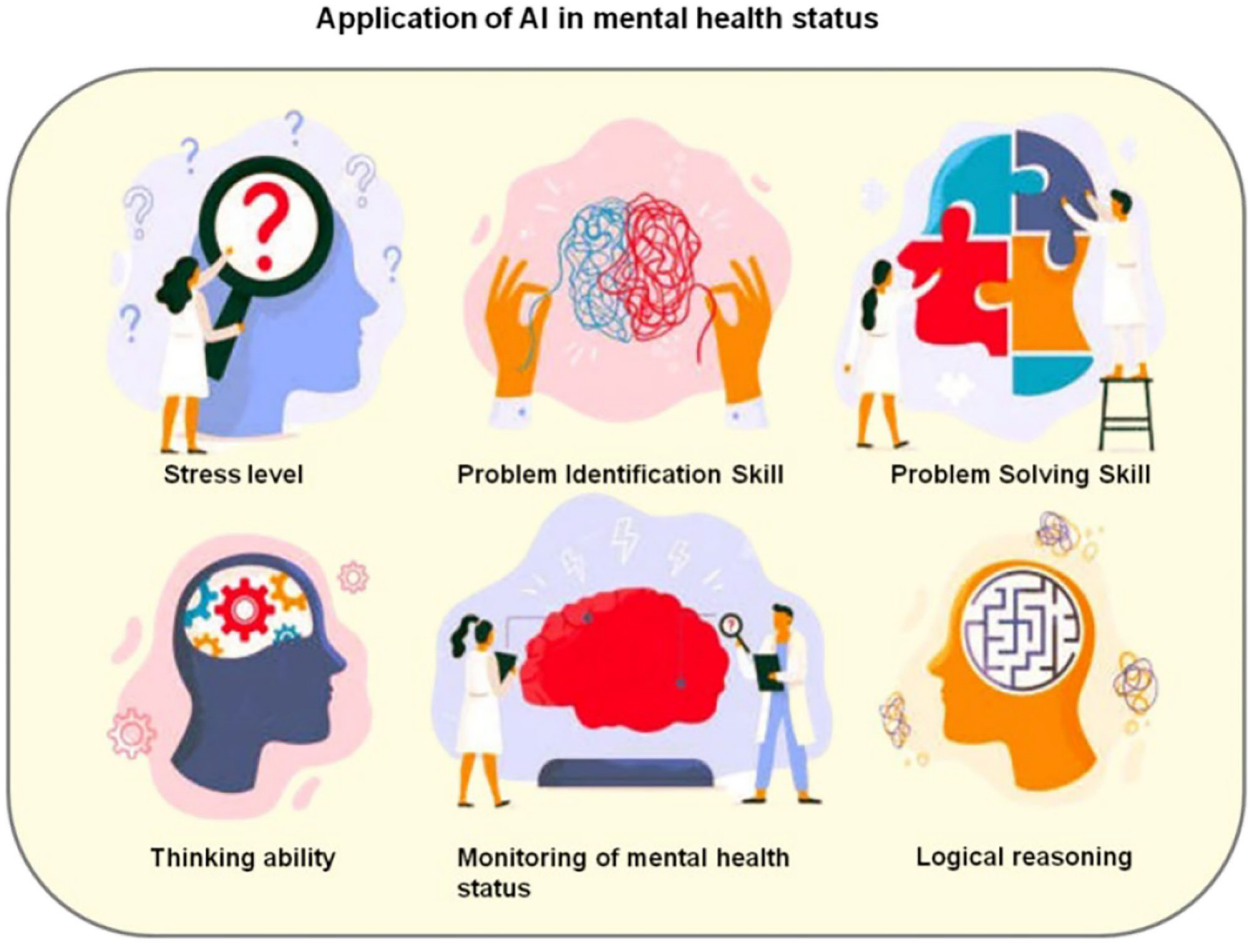
Application of AI in mental health status.

AI technology and methods are already in use all in our region, while frequently after the scenes. Many uses of AI technology and methods have grown so prevalent that we can no longer respect them to be AI-based. Artificial Intelligence-based mental health monitoring solutions ranging from smartphone apps to wearable gadgets may enhance or possibly replace the function of a psychologist as technology advances. It explains how AI can be used to make decisions and how it can be used to evaluate and treat patients. AI advancements are also helping the behavioral and mental health care industries (5). Figure 1 shows the application of AI to mental health status.

Computing tools for education, comprehending, and thinking, for example, may aid medical managerial, test, diagnosis, and care organization for healthcare practitioners. AI technology and approaches, such as participatory movable healthiness applications (apps) that learn users' behaviors and preferences, may develop self-care tools to get better people's lives. By aiding in identifying health concerns and guiding actions, AI is enhancing public health (6).

Despite the enormous development in academic output in computerization technologies, artificial intelligence, and robotics, academics still lack a clear understanding of the effects of these advances on human resource management (HRM) at both the managerial and personal levels. Consequently, the purpose of this study is to consolidate the current academic inputs on intelligent automation and characterize its key contributions to and issues with HRM (7).

Mental illnesses like depression are becoming more prevalent and have substantially impacted a person's physical well-being. AI systems have freshly been created to assist psychological well-being practitioners, especially psychiatrists and clinicians, and historical data from patients, such as clinical aspects, social media activity, and behavioral data, are used to make judgments. There is a pressing need to address adolescent core mental health concerns, which may progress to more severe problems if not recognized early (8).

AI platforms' computation capability might be used to elucidate the complicated biology of mental diseases and effectively guide treatment application. Effective physician relationships are vital in mental health treatment, but they are sometimes hampered by the little contact time allocated for medical care. AI technologies provide a method to automate activities that don't need a “personal contact,” allowing physicians to concentrate on providing more empathetic care and “civilizing” the tradition of medicine (9).

This research presents a novel CF-KNN approach to anticipate adolescents' mental health status by AI's application. The normalization technique is utilized to normalize/balance the collected datasets in the pre-processing stage. The VAE technique is employed to convert the normalized data into numerical attributes. The MG-ACO approach is performed to select the attribute sub-sets. The T-C4.5 and F-LRA techniques are conducted to assess the adolescent's mental health status, both t. The additional detail is organized as topic II-related works and problem statements, topic III-proposed work, topic IV-performance analysis, and topic V-conclusion.

## Study Background

This part illustrated the exciting approaches regarding legal knowledge and the prediction of adolescents' mental health status. AI technologies have been implemented to help physicians and doctors make decisions based on clinical information. There seems to be a great need to deal with fundamental psychological problems in youngsters that might become complex even though not addressed early. The deep learning assisted Integrated Prediction Model (DLIPM) was introduced in this research to detect and diagnose childhood mental disorders (8). Kubiak et al. (10) investigated the acceptance, faithfulness, and effects of a juvenile Crisis Intervention Team (CIT-Y). This strategy seeks to maintain mentally ill kids out of the criminal justice through educating and training police personnel. Singh et al. (11) examined the psychological well-being of young people affected by the COVID-19 virus, including statewide or local closings to limit illness propagation and during the COVID-19 epidemic; they reviewed papers and warnings on youth mental wellness.

Major psychological health is linked to physical ailments, but most study has been done on adults. To summarize available research on medical and psychological metabolic abnormalities among adolescent mental health inpatients. They executed the meta-analysis (12). A convenience sample strategy was used to deliver an online survey among kids and teenagers (13). A cascade technique enables assessing delivery difficulties and creating measures to increase treatment involvement and achievements for kids in community JJ supervision (14). Li's (15) study analyses the impact of music learning to enhance students' mental health in the setting of 5G networks. Based on the site study on the emotional and mental health of current academy students, one concerning the issue of music instructors seems to be how effectively to employ music education to assist students in maintaining a better and upward psychological condition. Zhang's (16) research proposes using triangular fuzzy and entropy weights to assess youths' mental well-being. This approach minimizes manual interaction and quantifies the outcomes of psychological screening amongst young adults. Cai (17) research presents a data mining-based approach to determining mental disasters in young adults to increase the accuracy of detecting mental disasters in young adults. A mental disorder recognition model based on CNN is developed in this research. Self-learning capability allows the model to detect mental illnesses in adolescents and assist academic psychologists and the mental well-being group (18).

Mody and Bhoosreddy (19) stated that many disorders have multiple odontogenic keratocysts. A 12-year-old female youngster had several odontogenic keratocysts. The studies found no other anomalies indicative of a condition. Personalized medicine employs fine-grained data to identify specific deviations from normal (20). These developing data-driven health care methods were conceptually and ethically investigated using ‘Digital Twins' within engineering. Physical artifacts were coupled using digital techniques which continuously represent their state. Moral differences can be observed based on their data structures and interpretations. Digital Twins’ ethical and sociological ramifications are examined. The Healthcare system has become increasingly data-driven. This technique could be a social equalizer by providing efficient equalizing enhancing strategies. Allergic rhinitis would be a long-standing worldwide epidemic. Taiwanese doctors commonly treat it with either traditional Chinese or Chinese–Western drugs. Outpatient traditional Chinese medicine therapy of respiratory illnesses was dominated by allergic rhinitis. They compare traditional Chinese medicine with western medical therapies in treating allergic rhinitis throughout Taiwan (21).

High-dose-rate (HDR) brachytherapy avoids radioactivity, allows for outpatient therapy, and reduces diagnosis timeframes (22). A single-stepping source could also enhance dosage dispersion by adjusting latency at every dwell location. The shorter processing intervals do not permit error checking, and inaccuracies could injure individuals. Hence, HDR brachytherapy therapies should be performed properly. Li (23) presented treatment and the technology of domestic sewage to improve the rural surroundings. Salihu and Iyya (24) took soil samples from chosen vegetable farms throughout Zamfara State. Nigeria has been tested for physicochemical and organochlorine pesticides. Testing procedure and data were analyzed using QuEChERS with GC-MS.

Rao et al. (25) mentioned that to prevent depression and enhance children's psychological levels, an “Artificial Neural Network-based Psychological Symptom Prediction Model” “(ANN-PSM)” has been suggested. In the presence of sad signals and an elevated risk of depression, the ANN-PSM patterns may identify youngsters who are more prone to perpetrate emotional abuse. Their studies highlight the processes that occur in children for social-emotional adaption due to mental depression.

AI is strongly linked to college students' ethical wisdom education as network technology advances in the internet era. It necessitates instructors' employment of AI and broadcast technology for college students to develop proper civic ethics via bright education approaches (26). The scoping review summarizes the present capabilities of AI-assisted digital biomarkers for dementia early on diagnosis and future potential research areas (27).

Li et al. (28) judiciously summarize psycho radiological studies using magnetic resonance imaging of the brain to investigate the neural mechanisms of psychiatric diseases and outline progress and the path ahead for the use of psychopathology and AI in sufferers with psychiatric disorders, as well as restrictions in the operation of AI that should be considered in destiny translational research.

Welch et al. (29) performed a scoping review to explore, define, and field wearable technology discovery that may supplement existing in-person physician evaluations to personalize treatment and care of mental problems in children and adolescents. Malek (30) investigated how and why AI technologies promote prejudice and inequality in society and possible alternatives to present predictive justice methods.

The back propagation (BP) algorithm is used in this investigation study to diversity various features from past data, like psychological character, fundamental individual aspects, and social-economic factors. Data grounding techniques are used in the early stages of modeling to construct the data for use by the BP algorithm to create the portrait. The capability of the BP algorithm to knob variability in data and explore associations between numerous attributes is one of the reasons for its acceptance (31).

### Problem Statement

Law enforcement can now analyze young adults' capacity and legality. Knowing that institutions have sensible laws allows students to research health and life advancement appropriately. They chose legally possessed firearms to protect themselves and other students from assaults. Law students were able to learn and achieve together to raise legal awareness, fight against rules and infractions, and implement the nation's efficient legal guidance. For this, several investigations are progressed but with specific inconsistent outcomes in learning efficiency, students' mental ability prediction, etc. We introduce a novel cognitive fuzzy-based K-nearest neighbor (CF-KNN) by AI application to legal education students to overcome these concerns.

### Proposed Work

**Figure 2:**
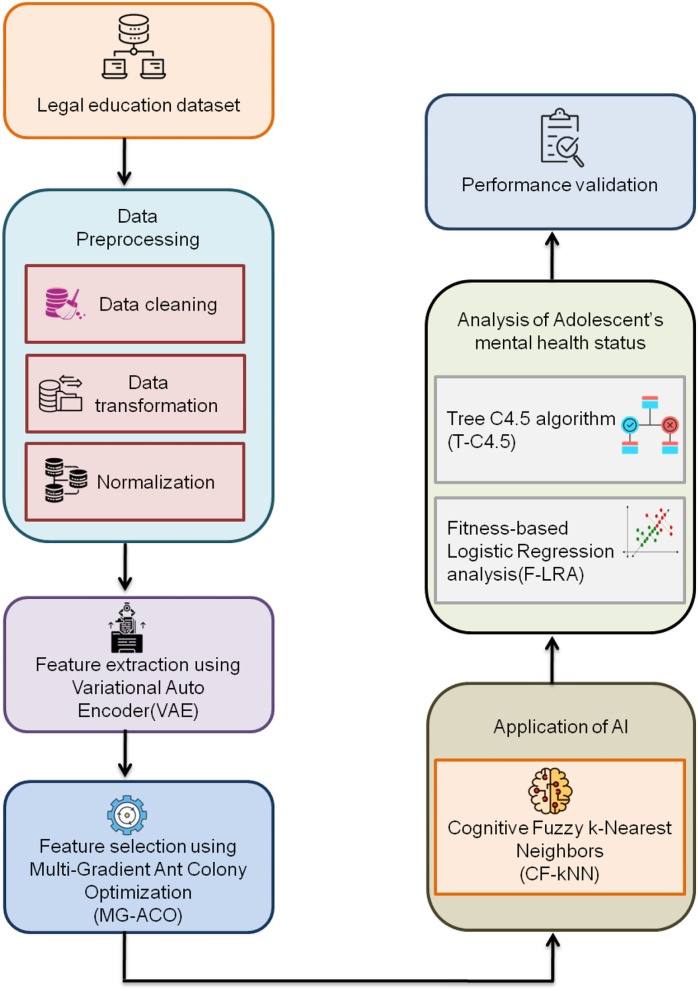
Complete framework of proposed work.

This research aims to evaluate the impacts of AI on the legal field and the mental health conditions of adolescents. For this purpose, the cognitive fuzzy K-nearest neighbor (CF-KNN) technique is utilized. Specific legal education datasets are gathered to initiate this investigation. A normalization approach is provided to pre-process these datasets to eliminate the unwanted noises/outliers from the gathered raw data. Correspondingly, feature extraction and feature selection stages are performed through VAE and MG-ACO techniques. T-C4.5 and F-LRA techniques are used to assess the adolescent's mental health conditions. Furthermore, the performance of our proposed work is examined to prove our work with better outcomes. Figure 2 depicts the complete framework of this research.

## Study Dataset

This research is carried out within Fuyang Township, Guangdong Province, from Dec 2007 to June 2008 (32). Fuyang has a population of 87,134 and per capita yearly revenue of around 5,154 RMB (2008), which is also under the national and province averages of 4,140 RMB and 5,624 RMB, correspondingly. It's a category II rural area in the municipality (category I am extremely rich, and category IV is extremely deprived). Thirteen to 18-year-olds can attend any of the five junior high schools (1 vocational and 4 standards) or the senior high schools that serve students between the ages of 16 to 18. The research was open to all primary and secondary education students in grades one and two. In advance, the pupils were asked for their written agreement, which was received voluntarily. As a result, we were given the go-light by CHU's Ethics Committees. Table 1 shows the dataset description.

**Table 1:** Study respondent.

**Sl.no**	**Variables**	**Descriptions**
1	Province	Fuyang Township
2	Year	December 2007 to June 2007
3	Population	87.134/capita revenue around 5,154 RMB (2008) National and province averages of 4,140 RMB and 5,624 RMB
4	Age of response	13–18 years

### Data Pre-processing

Quality data is still an issue, and poor information may result in bad judgments and analyses. Typical problems are missing values, errors, confusing forms, duplicate records of the same real-world entity, and medical guideline breaches. To make informed decisions, we should evaluate the impacts of dirty data. Missing values become significant because these alter the information's properties. The values should be calculated to do a dynamic analysis, mainly whenever numerous studies are involved. It can cause serious identification issues. Data corruption causes discontinuity in data records or outliers, and thus it indicates uncertainty in the identification process.

So, that data must be removed containing missing values and outliers. Therefore, data pre-processing seems to be a critical step in obtaining resultant data that are accurate and valuable for more investigation. The dataset supplied is unrefined and will include a bogus datagram and insufficient data. It has been cleaned and standardized to remove recurrent and duplicate sounds and insufficient data. Due to the huge size of the records, specimen compression procedures must be used. Because there are so many characteristics in this dataset, image retrieval techniques are required to filter out the ones that aren't important.

The dataset may be normalized during the pre-processing stage. Equation (1) defines the q-count in mathematical form as,

(1) $$\begin{array}{r} \text{q}=\left[\left(\text{B}-\beta \;\right)/\tau \right] \end{array}$$

Here, β expresses the mean of the information and τ hints at the standard deviation (33), and q is represented as,

(2) $$\begin{array}{r} \text{q}=\frac{\text{B}-\bar{\text{B}}}{\text{Sd}} \end{array}$$

Here, *B* = mean of the specimen, and Sd points out the standard deviation of the specimens.

This is how the random specimen looks like:

(3) $$\begin{array}{r} q_{k}=\delta_{0}+\delta_{1}B_{r}+\rho_{r} \end{array}$$

The defects that are depending on τ^2^ are represented by r.

Ensuring that, as seen below, the defects should not depend on one another.

(4) $$\begin{array}{r} t_{m}\sim \sqrt{U}\frac{t}{\sqrt{t^{2}+u-1}} \end{array}$$

Here, the t = random parameter.

After that, the standard deviation is used to standardize the variable's moves. The momentary scale deviation is calculated using the formula (5).

(5) $$\begin{array}{r} \text{MMS}=\frac{\mu^{\text{mms}}}{\theta^{\text{mms}}} \end{array}$$

Here, momentary scale = mms.

(6) $$\begin{array}{r} \mu^{\text{mms}}=\text{Ex}\left(\text{B}-\beta \right)ˆ\text{MMS} \end{array}$$

Here, B = random variable, and Ex = predicted values.

(7) $$\begin{array}{r} \theta^{\text{mms}}=\left(\sqrt{\text{Ex}\left(\text{B}-\beta \right)ˆ\text{MMS}}\right)ˆ2 \end{array}$$

(8) $$\begin{array}{r} t_{u}=\frac{mms}{\bar{B}} \end{array}$$

The coefficient of variance = *t*_*u*_.

By setting all of the parameters to 0 or 1, the characteristic scaling method will be terminated. This process is known as the unison-based normalizing approach. This is how the normalized formula would look:

(9) $$\begin{array}{r} B^{\prime }=\frac{\left(t-t_{\min}\right)}{\left(t_{\max}-t_{\min}\right)} \end{array}$$

After the data has been normalized, the statistics may be retained, and the length and irregularity of the data can be preserved. The goal of this step is to reduce or eliminate information repeats. After that, the normalized data may be utilized for further processing.

### Feature Extraction Using Variational Autoencoder

VAE is a deep Bayesian architecture that incorporates neural network models and analytics. It also forces the underlying signals to pursue a specified allocation, like the Gaussian distribution, instead of the typical auto-encoder. This change improves the learned aspects' ability to meet the demands of our target.

**Figure 3:**
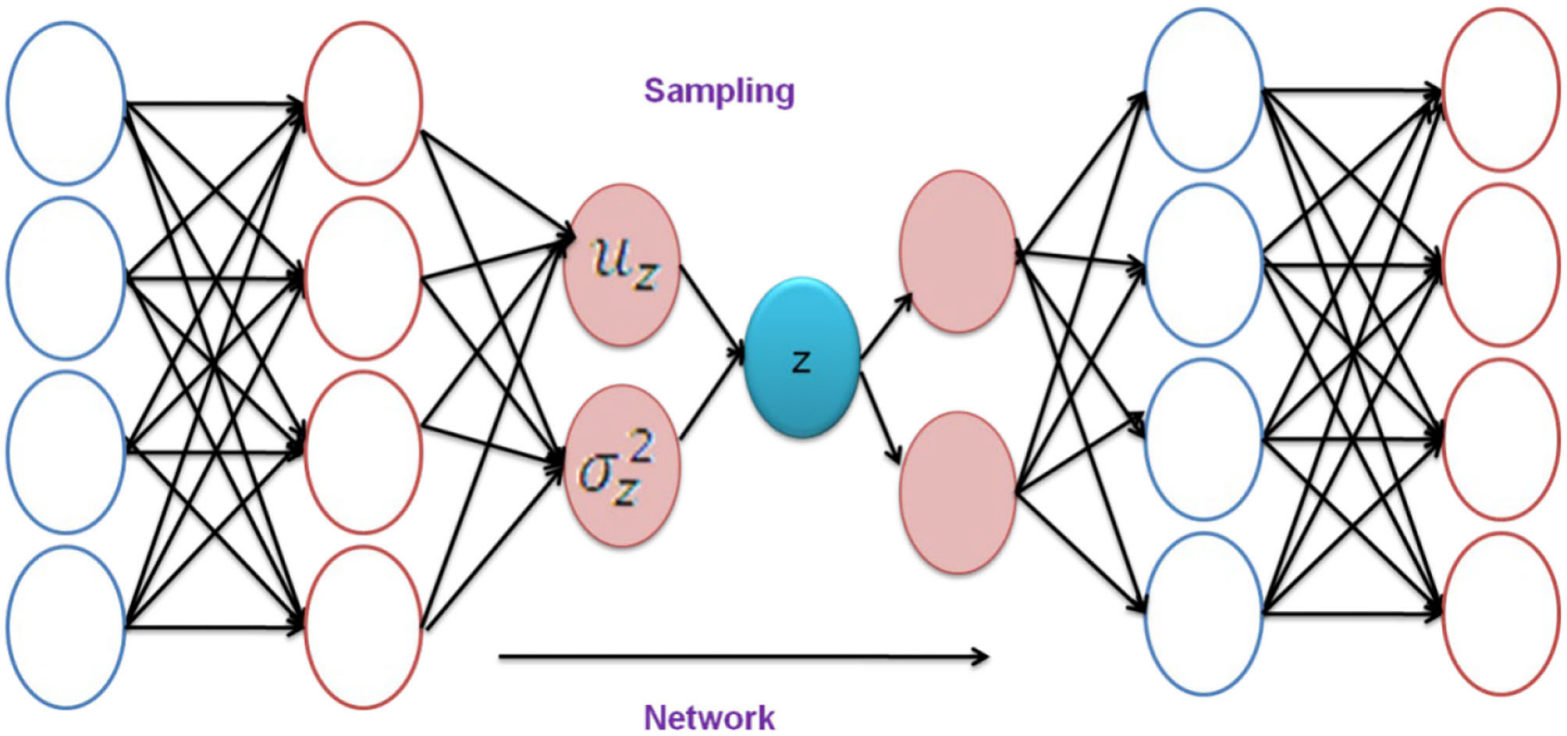
Framework of VAE.

The VAE's entire layout is depicted in Figure 3. The fundamental principle of VAE is introduced in this phase. The encoder component of the neural network is supposed to show the conditional probability as,

(10) $$\begin{array}{r} E_{t}=\left(u|f\right) \end{array}$$

Here, f = authentic information; t = encoder's weight; u = inherent codes.

Dissimilar to other auto-encoders, the VAE pushes the underlying codes “u” dispersion to resemble a conventional normal distribution. The underlying codes have persistent analytical features, making the decoder easier to use and our operation more efficient. The decoder then uses the underlying codes to obtain the model information. A neural network with weight, for example, “γ” indicates a conditional probability,

(11) $$\begin{array}{r} P_{\gamma }=\left(f|u\right) \end{array}$$

A new VAE technique must be implemented. The decoder's sources are taken from the distribution of underlying codes to maintain ambiguity within the networks. The encoder is then programmed to produce two factors: the underlying codes' average and deviation vector. The two factors are then used to create a normal distribution to select a signal for the decoder.

### Feature Selection Using Multi-Gradient Ant Colony Optimization

**Figure 4:**
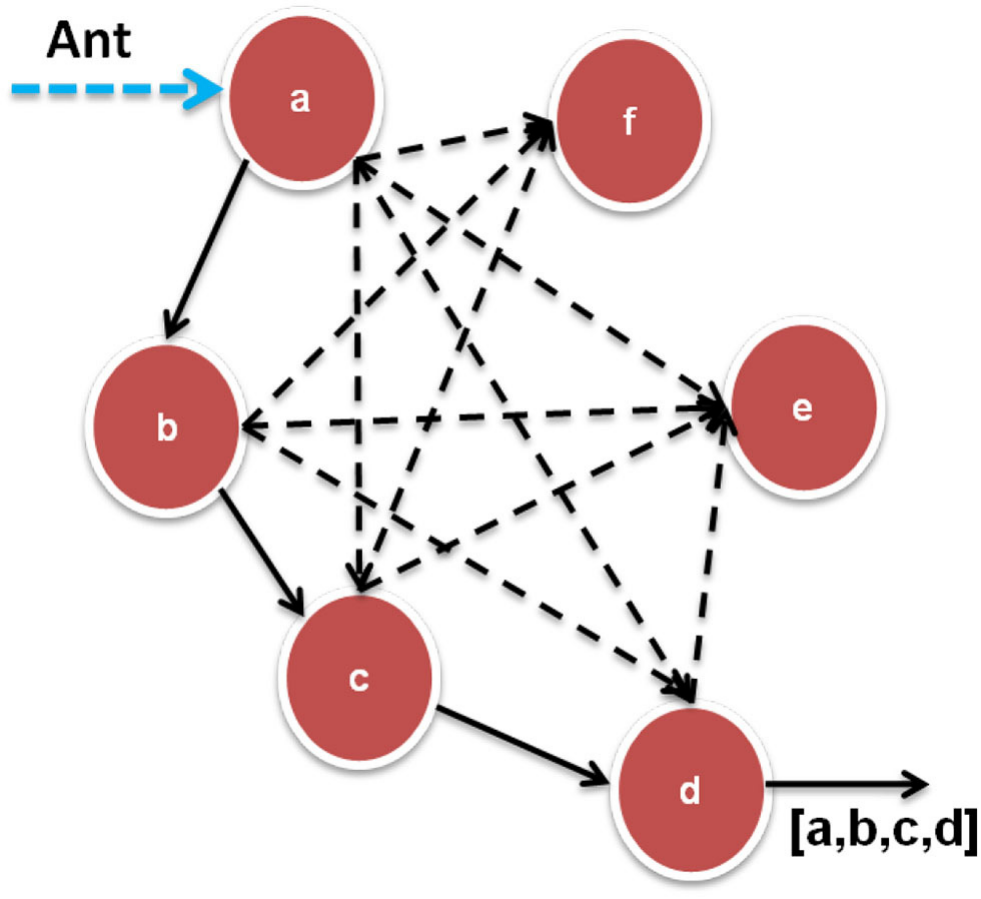
Layout of MG-ACO problem.

The feature selection challenge could be framed as an MG-ACO issue. An issue must be represented graphically in MG-ACO as depicted in Figure 4. The edges between nodes reflect the decision of another characteristic, while the nodes themselves indicate characteristics. The search for the perfect feature subset proceeds like an ant transit through the structure, terminating when the smallest nodes are visited to meet the traverse terminating condition. The ant gets presently at node ‘a’ and can add which characteristic to its pathway ahead (dotted lines). Depending on the transition criterion, it selects characteristic b next, following c, and finally d. The present subset [a; b; c; d] gets evaluated to fulfill the traverse termination requirement when arriving at d. The ant finishes its traverse and delivers this characteristic subset as an information reduction candidate.

Any subset assessment task, such as an entropy-based measurement, a rough set dependency test, or FDR (“Fisher Discrimination Rate”), will be used as an appropriate heuristic for transiting among characteristics. The probabilistic transition criterion, which denotes the probability of an ant at characteristic “i” deciding to move to feature j at period t, is formed by combining the heuristic attractiveness of traversal with edge pheromone amounts.

(12) $$\begin{array}{r} p_{ij}^{k}\left(t\right)=\{\begin{array}{c} \frac{{\left[\tau_{ij}\left(t\right)\right]}^{\alpha }\cdot {\left[\eta_{ij}\right]}^{\beta }}{\sum_{l\epsilon J_{i}^{k}}{\left[\tau_{ij}\left(t\right)\right]}^{\alpha }\cdot {\left[\eta_{ij}\right]}^{\beta }}\;if\;j\epsilon J_{i}^{k}\\ 0\;otherwise \end{array} \end{array}$$

Here, k = no. of ants, η_*ij*_ = heuristic desirability of selecting feature “j” while at feature “i”, $J_{i}^{k}$ = collection of neighbor nodes of node “i” that the ant has not yet explored, and τ_*ij*_(*t*) = quantity of virtual pheromone present upon that edge (i,j).

And also, the proportional significance of the pheromone score and heuristic information was determined by two variables: alpha and beta, and values of both are greater than zero.

**Figure 5:**
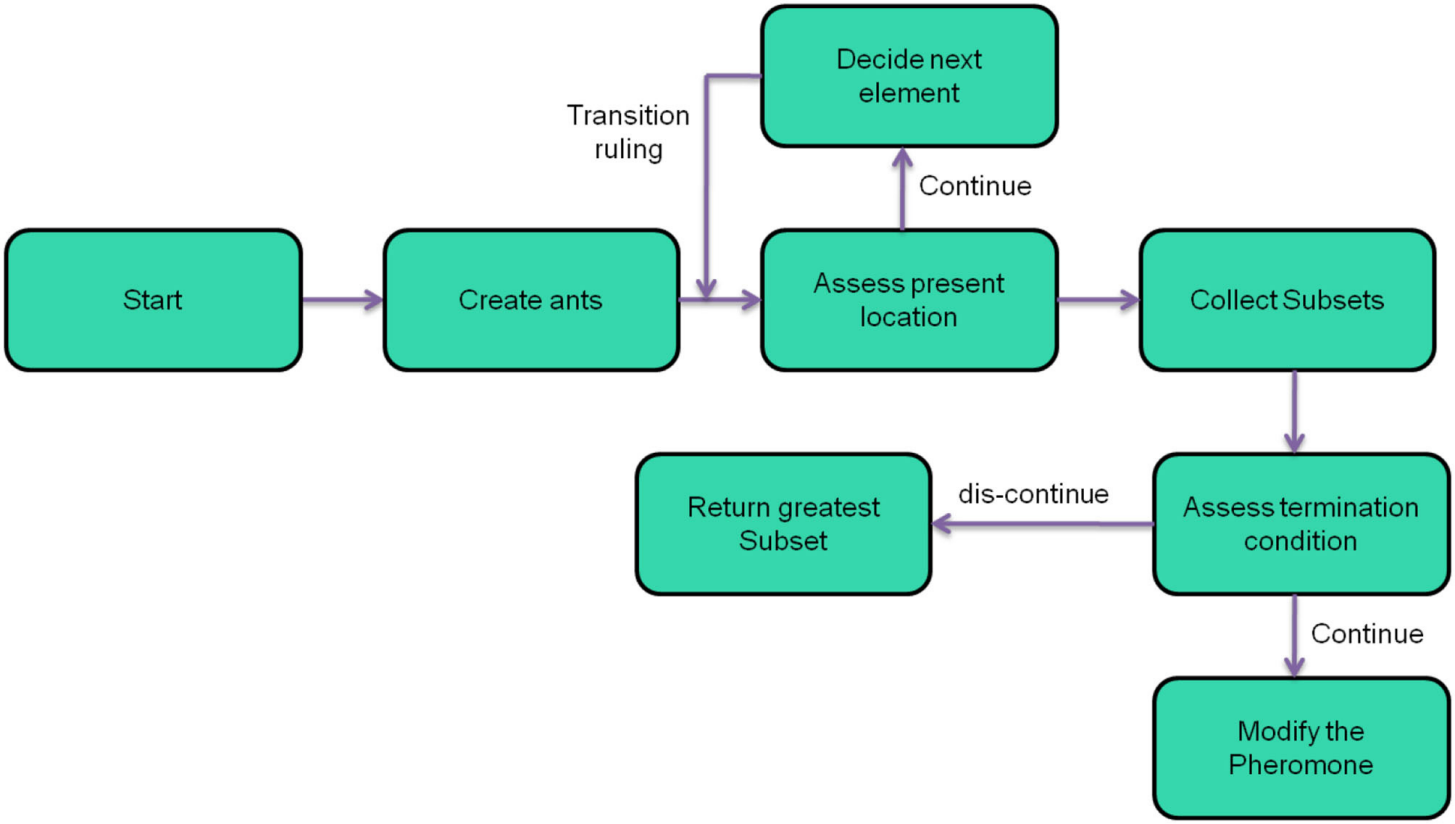
Procedure of MG-ACO.

Figure 5 depicts the process flow of MG-ACO. The procedure starts with generating a great number of ants, k, which would then be arbitrarily arranged upon that graph. Instead, the number of ants to arrange on the graph could be specified as the same number as the number of features inside the data, with every ant initiating pathway creation at a distinctive characteristic. They traverse edges based on probabilities from such starting places until a traversal termination requirement is fulfilled. The subsets that arise are then collected and assessed. The procedure stops and delivers the greatest feature subset observed even when an excellent subset was already discovered, or the technique has run a given number of times. If neither of these conditions is met, the pheromone was adjusted, a new group of ants is formed, and the cycle continues.

The equation for updating the pheromone on every edge is represented as:

(13) $$\begin{array}{r} \tau_{ij}\left(t+1\right)=\left(1-\rho \right).\tau_{ij}\left(t\right)+\rho .\Delta \tau_{ij}\left(t\right) \end{array}$$

Where

(14) $$\begin{array}{r} \Delta \tau_{ij}\left(t\right)=\overset{n}{\underset{k\;=\;1}{\sum }}\left(\frac{\overset{´}{\gamma }\left(S^{k}\right)}{\left|S^{k}\right|}\right) \end{array}$$

If indeed the edge (i;j) was already visited, Δτ_*ij*_(*t*) represents 0; alternatively, ρ = decay constant has been used to mimic pheromone dispersal within the range [0, 1], and S^k^ = feature sub-set discovered by ant ‘k’. The pheromone is modified depending on the size of the ant's feature subset (γ′) and the assessment of its quality. Every ant modifies the pheromone through this concept.

### Cognitive Fuzzy K-Nearest Neighbor

Standard Euclidean distance “D (a, b)” has been utilized to estimate the difference between the training and test examples. The following equation calculates it:

(15) $$\begin{array}{r} D\left(a_{i},\;a_{j}\right)=\sqrt{\sum {\left(m_{r}\left(a_{i}\right)-m_{r}\left(a_{j}\right)\right)}^{2}} \end{array}$$

To assess the class of a training example in the testing dataset, KNN calculates the most common type of K closest neighbor to the test data. The following equation is described:

(16) $$\begin{array}{r} c\left(a\right)={\arg\max}_{c\;\in \;C}\overset{k}{\underset{i\;=\;1}{\sum }}L\left(c,\;c\left(b_{i}\right)\right) \end{array}$$

Here, k = amount of neighbors, y_k_ = K^th^ nearest neighbor of a particular test instance of the test dataset, C = finite set of class labels and *L*(*c, c*(*b*_*i*_)) is equal to “one” when c = c(b_i_) and *L*(*c, c*(*b*_*i*_)) is equal to zero in other cases.

**Figure 6:**
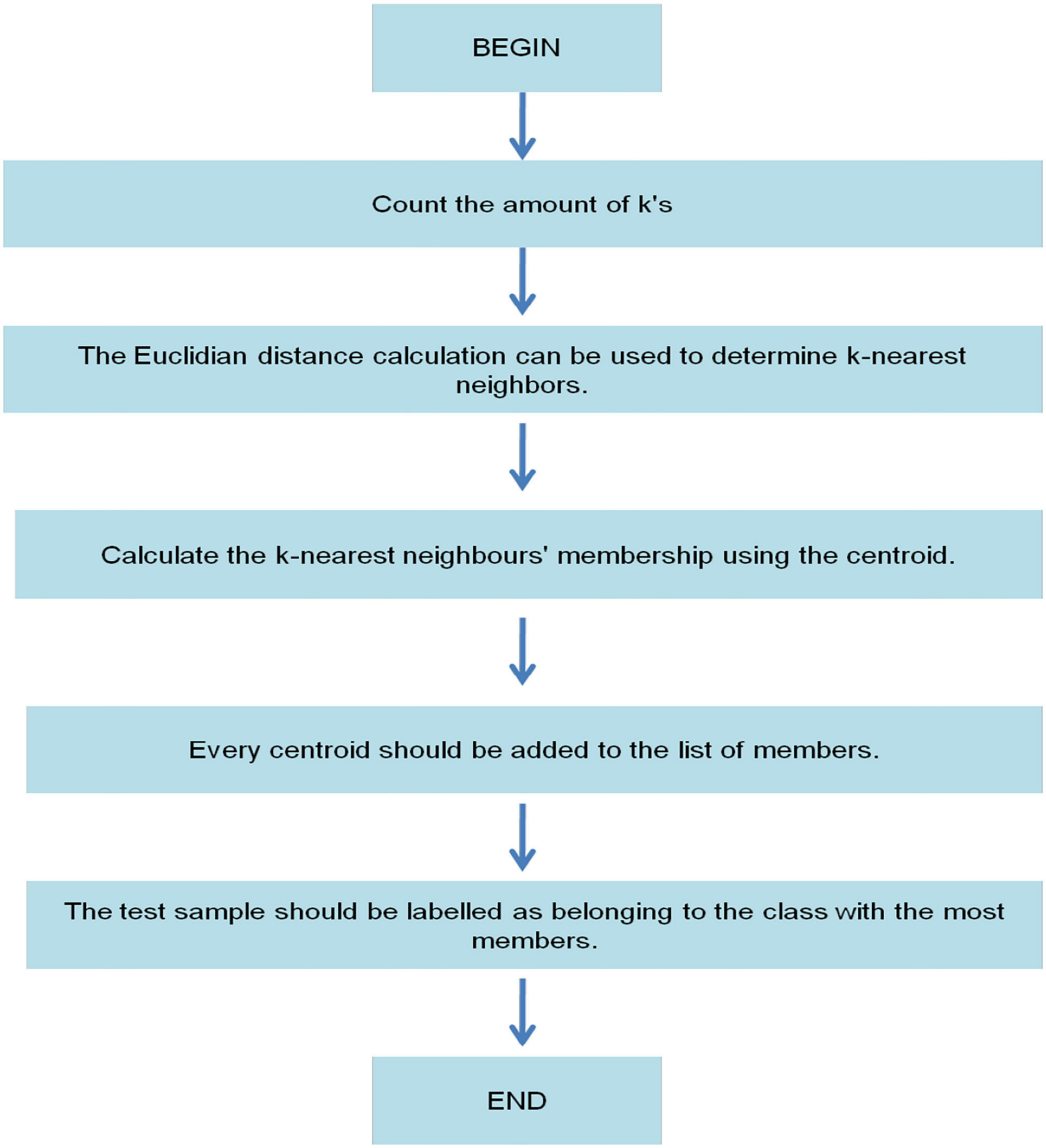
Flow process of CF-KNN.

When using the KNN classifier, one of the biggest issues is that every sampled data is given equal weight when assigning a class label to the raw data. This creates an issue if the samples are too close together. When an input sample has been granted a class label, there is no way to tell how strong it belongs to that particular class. CF-KNN solves two problems with the traditional KNN algorithm. The CF-KNN exceeds the more classic KNN approach as a mere fuzzy version. Algorithm 1 depicts the procedure, and Figure 6 depicts the flow process of the CF-KNN technique.

**Algorithm 1:** Procedure of CF-KNN.

**Stage-1:** Upload the dataset
**Stage-2:** Using the KNN method, identify the closest neighbors of every test piece.
**Stage-3:** Add the KNN's centroid to the list of members.
**Stage-4:** If a centroid has a high degree of membership, use that centroid's class label for determining this sample.

### Tree C4.5 (T-C4.5) Algorithm

There are nodes and edges inside a resolution tree (DT). It is an element of the DT procedure employed in supervised learning. The development of the decision tree and the construction of the guidelines is the two primary components of the C4.5 overall (arrangement and intent). Determine the entropy and gain for the feature that has the most value in the selection.

A decision tree can be generated using the Tree C4.5 technique in 4 stages. The feature is the first root. A branch is generated for each value. Finally, add the database to the branch. Fourth, keep repeating the second step when the same value is found in each class.

(17) $$\begin{array}{r} Entropy\;\left(R\right)=\overset{n}{\underset{i\;=\;1}{\sum }}-w_{i}^{*}{\text{log}}_{2}w_{i} \end{array}$$

Here, R = entropy and w = percentage in the output.

This is followed by using a root feature with a large gain rate.

(18) $$\begin{array}{r} Gain\left(C,F\right)=\;Entropy\left(R\right)\overset{n}{\underset{i\;=\;1}{\sum }}{\frac{\left|C_{i}\right|}{\left|C\right|}}^{*}Entropy\left(R\right) \end{array}$$

Here, C = collection of cases, F = feature of the case, |C| = amount of cases in the collection, and |Ci| = amount of cases to “i”.

As seen in Algorithm 2, the pseudo-code for Tree C4.5 can be found there.

**Algorithm 2:** Procedure of T-C4.5.

**Need:** a feature-appreciated database (d), Tree (T)
**Step-1:** If d is “free of contaminants” or other requirements are satisfied to prevent further progress, then the operation should be halted.
**Step-2:** End if
**Step-3:** For all features F ∈ d do
**Step-4:** If we divided upon F, calculate the information-theoretic criterion
**Step-5:** End for
**Step-6:** F_best_ = best feature as per the above-determined criterion
**Step-7:** T = generate a decision node that evaluates the F_best_ in the root
**Step-8:** d_v_ = create sub-sets or d depending upon F_best_
**Step-9:** For all d_v_ do
**Step-10:** T_v_ = C4.5 (d_v_)
**Step-11:** Connect T_v_ to the related T branch
**Step-12:** End for
**Step-13:** Return T

### Fitness-Based Logistic Regression Analysis

The primary goal of a fitness function would be to examine the effectiveness of every solution that is put out for consideration. Regression representations were tested using the outcome from F-LRA:

(19) $$\begin{array}{r} H\left(z\right)=\frac{h^{{}^{\prime }}}{1+h^{{}^{\prime }}} \end{array}$$

Where z = 1.

Here, H(z) = probability and z = 1.

(20) $$\begin{array}{r} g=\beta_{0}+\beta_{1}u_{1}+\beta_{2}u_{2}+\ldots \ldots ..\beta_{k}u_{k} \end{array}$$

Here, the cognitive test values are expressed in the cognitive test values, the g = linear function of explanatory factors, u = number of independent feature/variables, and that variable.

The AUC (“area under the ROC curve”) has been employed as a fitness metric in this experiment. Whenever it comes to the selection of features, this fitness function does a good job. However, it prefers to pick bigger sets of features. For this investigation, fewer variables in the study were chosen to obtain prediction performance and are comparable to those of the bigger sets. As a result, fewer variable collections are more useful since they are simpler to understand and evaluate. Smaller feature collections could be gathered more regularly because they are simpler, quicker, and far less expensive.

A penalty is added toward the fitness function to make it easier to identify limited selected features that seem to be significantly predictive:

(21) $$\begin{array}{r} fitness=AUC+q-\frac{qr}{m} \end{array}$$

Here, r = amount of active variables, m = amount of features, and q = compromise element.

It is achievable to diminish the numeral of variables by adjusting the ROC fitness offsets, which we're talking about here.

## Performance Analysis

In this section, the investigation of our work is explained, and certain performance metrics like learning interest, learning accuracy, sensitivity, specificity, and error rate are also assessed through the below-mentioned calculations and indicate the efficacy of the planned technique for the prediction of student's mental health status by applying AI. These metrics are depicted as outcomes using the MATLAB tool in the graphical representations. The legal education student's data are taken as datasets in this research. The pre-processing stage is carried out to standardize/balance the given datasets through normalization techniques to remove unwanted outliers/noises. The normalized data is performed in further processes like feature extraction, feature selection, and AI application to anticipate the psychological health status of legal education students. Here, the proposed technique is used to predict the mental health status of legal students. Our proposed technique is performed and also matched with other standard techniques such as BP-neural network (34), ANOVA (35), and Bayesian-BP branch (36), and VGG-19 (32). The metrics mentioned above are determined with both the proposed and standard techniques.

A student's mental health status can be classified as either a positive or negative event. We divided the dataset into four trials [i.e., “true positive,” “true negative,” “false positive,” and “false negative”]. Our proposed algorithm forecasts the data individually; therefore, we arrange them as per their prediction findings and then use the data as good instances. In this section, certain metrics like learning interest, learning accuracy, sensitivity, specificity, and error rate are assessed for our proposed work for predicting the student's mental health status. These metrics are expressed below.

### Learning Accuracy (A)

**Figure 7:**
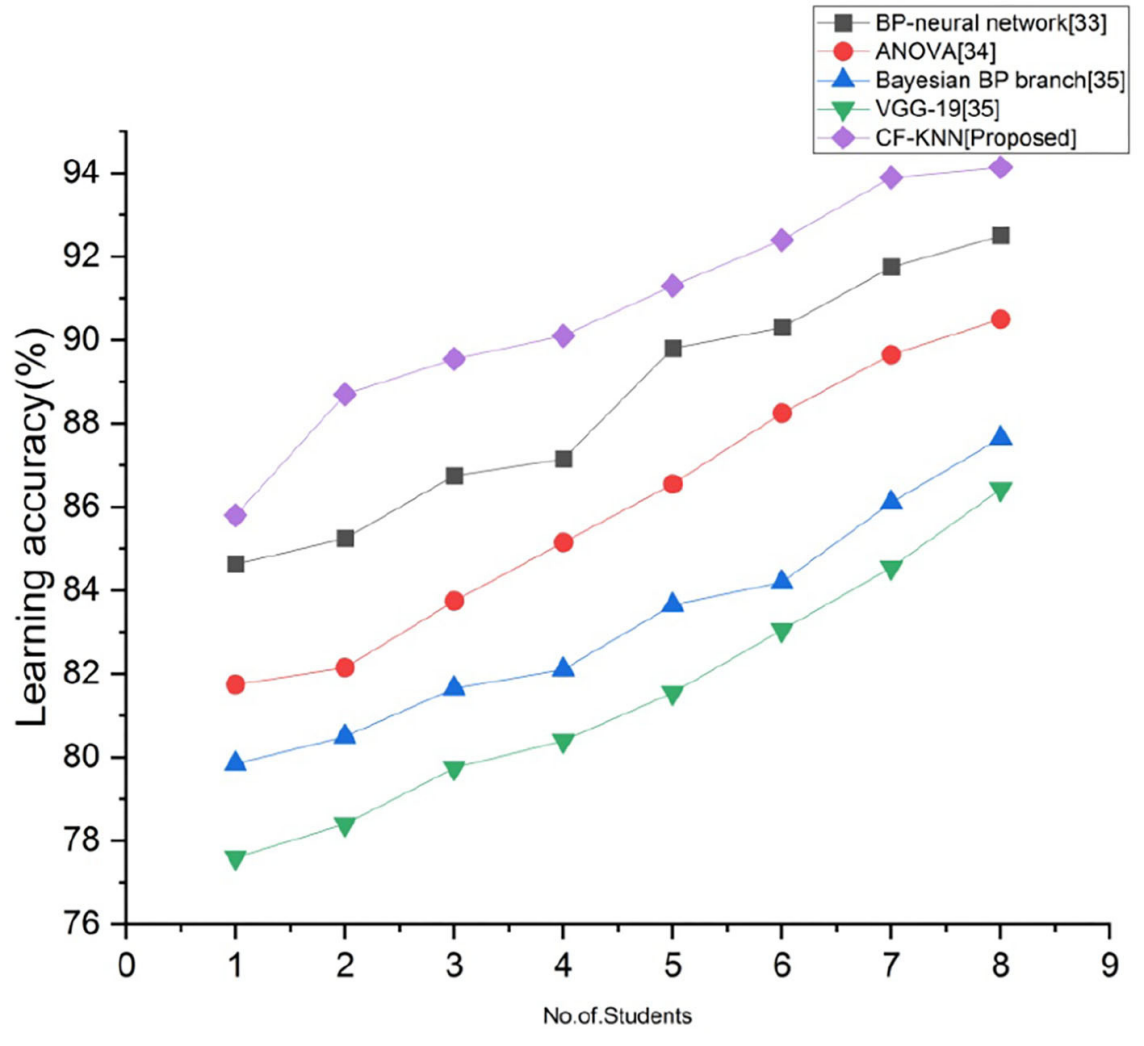
Comparison of learning accuracy with proposed and existing techniques.

Learning accuracy provides the categorization with the required information of the health status. Figure 7 depicts the comparison of learning accuracy with proposed and existing techniques for predicting students' mental health status by applying the AI model. By employing the below calculation, we accomplish the greatest learning accuracy than the existing techniques.

(22) $$\begin{array}{r} A=\frac{\left(T+U\right)}{\left(T+U+V+W\right)} \end{array}$$

Here, true positive = T = amount of right forecasts of a positive sample, true negative = U = amount of right forecasts of a negative sample, false positive = V = amount of wrong forecasts of a positive sample, and false negative = W = amount of wrong forecasts of a negative sample.

### Specificity (B)

**Figure 8:**
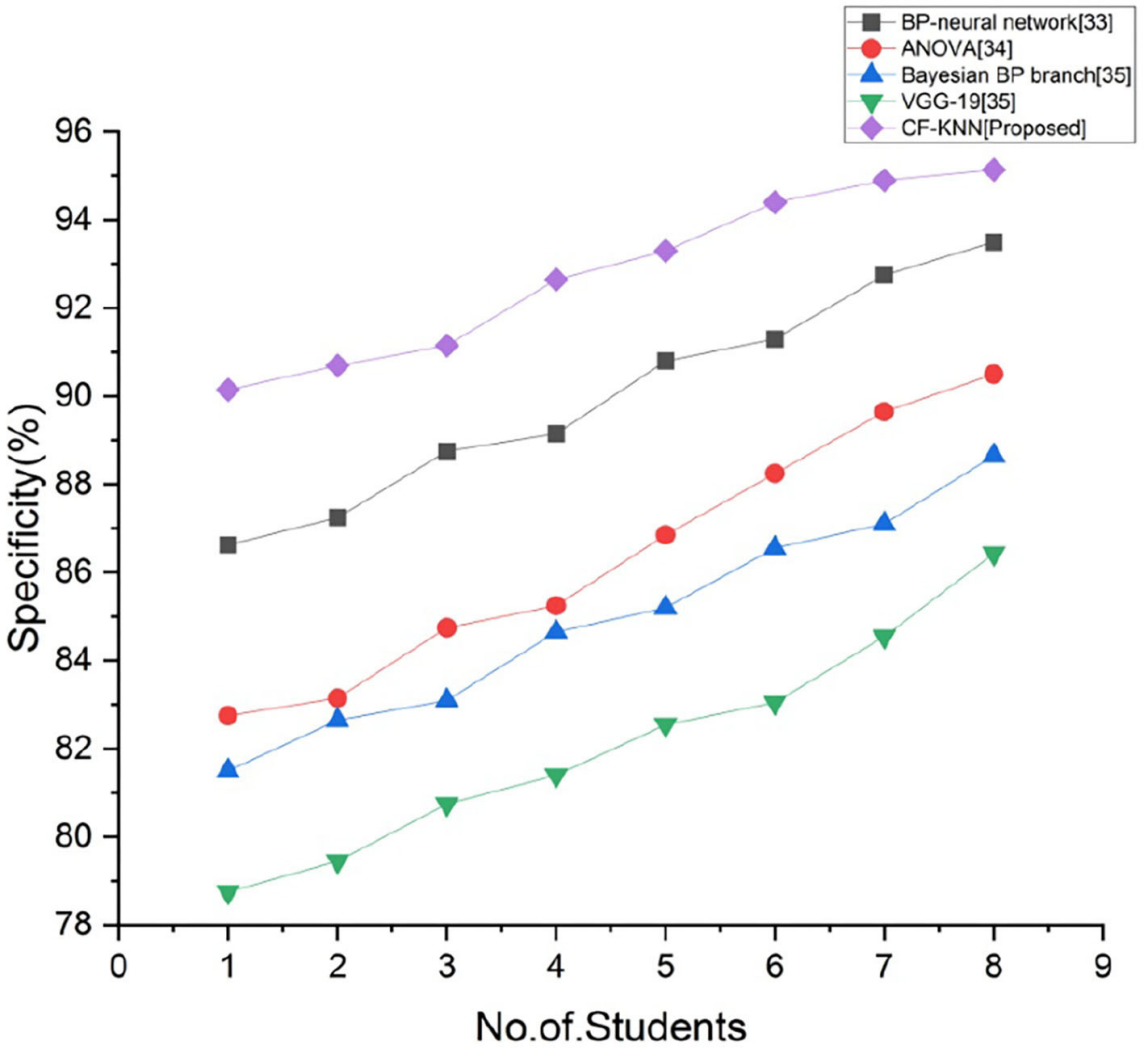
Comparison of specificity with proposed and existing techniques.

The effectiveness of a method to forecast the true negatives of every specified student's health status is measured by its specificity. Figure 8 depicts the comparison of specificity with proposed and existing techniques for predicting students' mental health status by applying the AI model. By employing the below calculation, we accomplish the greatest specificity of the existing techniques.

$$\begin{array}{l} B=\frac{U}{U+V} \end{array}$$

### Sensitivity (C)

**Figure 9:**
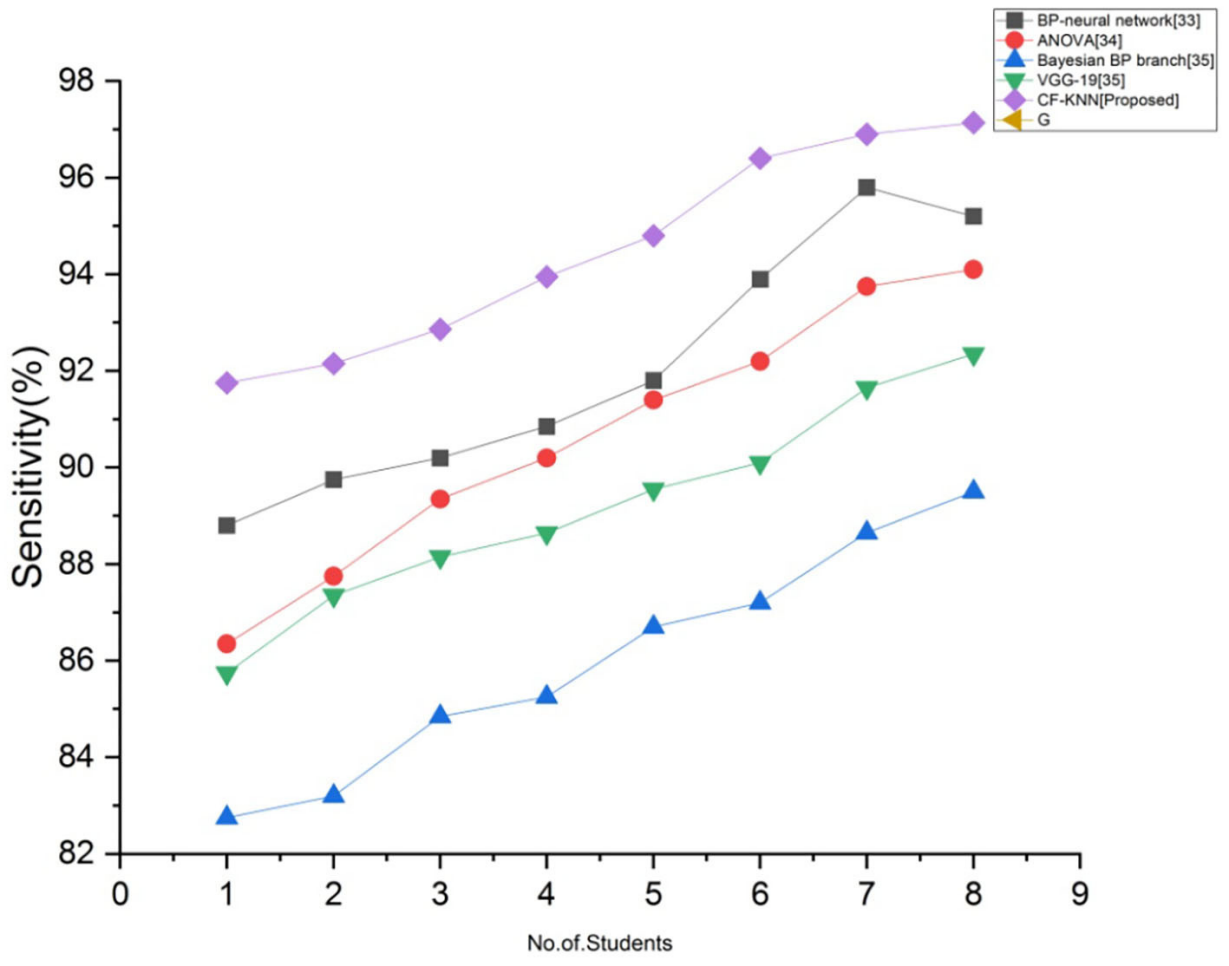
Comparison of sensitivity with proposed and existing techniques.

Sensitivity is the statistic that measures a model's potential to anticipate a true positive in every specified student's health status. Figure 9 depicts the comparison of sensitivity with proposed and existing techniques for predicting students' mental health status by applying the AI model. By employing the below calculation, we accomplish the greatest sensitivity than the existing techniques.

$$\begin{array}{l} C=\frac{tp}{tp+fn} \end{array}$$

### Error Rate (D)

**Figure 10:**
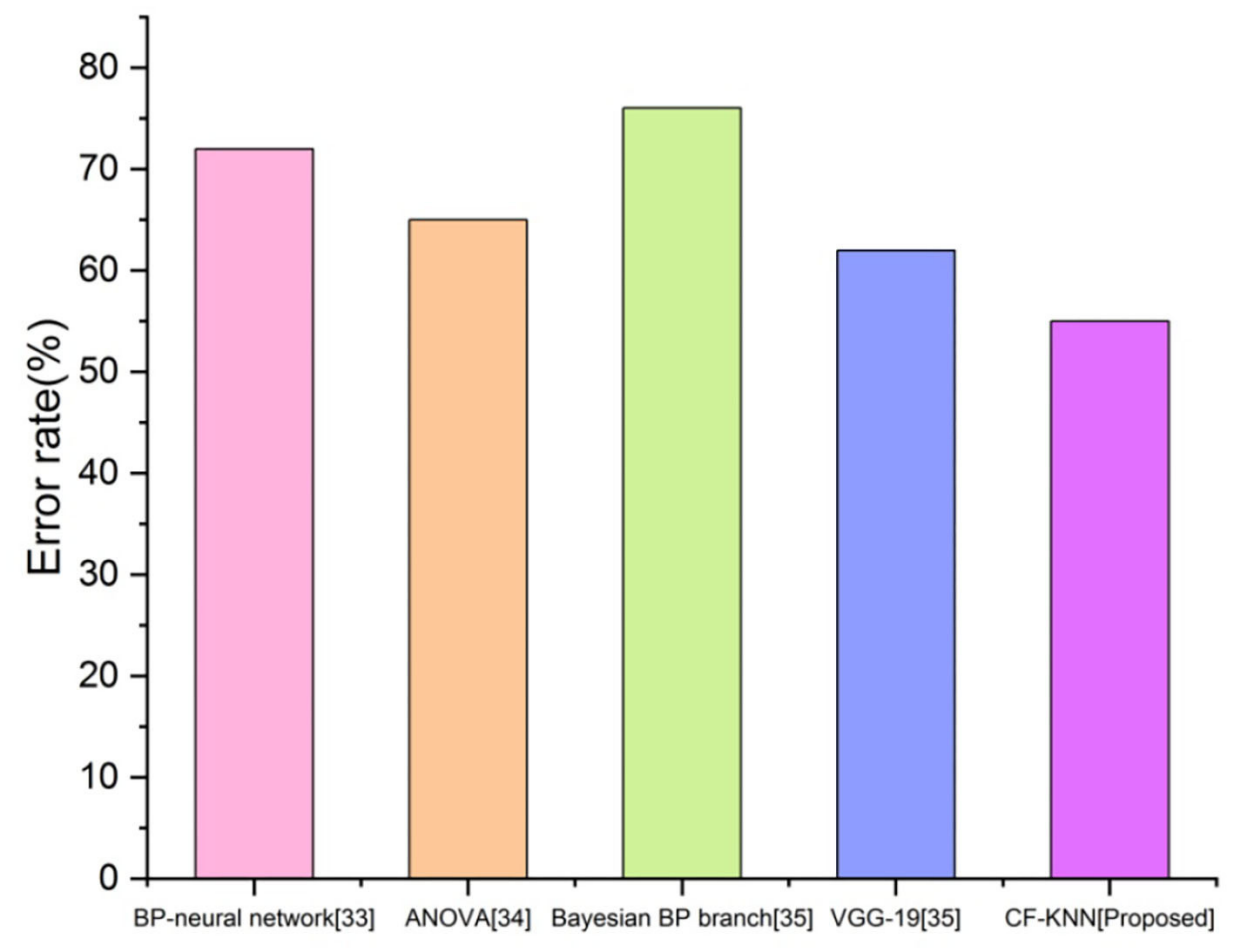
Comparison of error rate with proposed and existing techniques.

The error rate seems to result from incorrectly anticipated outcomes and is represented in percentages. Figure 10 depicts the error rate comparison with proposed and existing techniques for predicting students' mental health status by applying the AI model. By employing the below calculation, we accomplish the smallest error rate than the existing techniques.

$$\begin{array}{l} D=\frac{measured\;value-predicted\;value}{\;predicted\;value}\times 100 \end{array}$$

### Learning Interest

**Figure 11:**
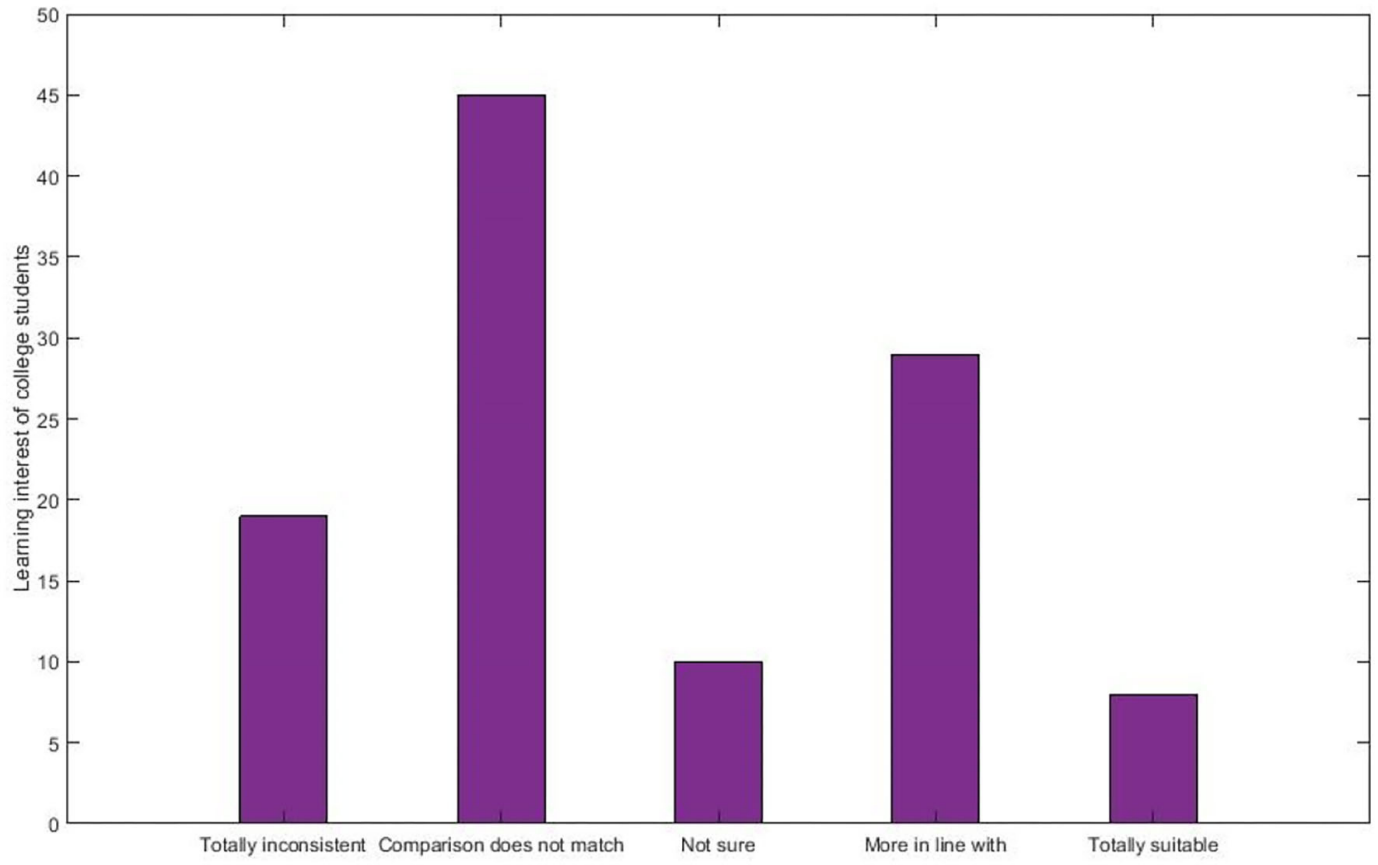
Learning interest.

Students' interest in AI is defined in learning as their ability to interact with that subject matter without any difficulty readily. Figure 11 depicts the student's learning interest in the proposed technique for predicting a student's mental health status by applying the AI model. By employing the below calculation, we get students' learning interest rate in different cases like “totally inconsistent. The comparison doesn't match, not sure, more in line with, totally suitable.”

### Efficiency

**Figure 12:**
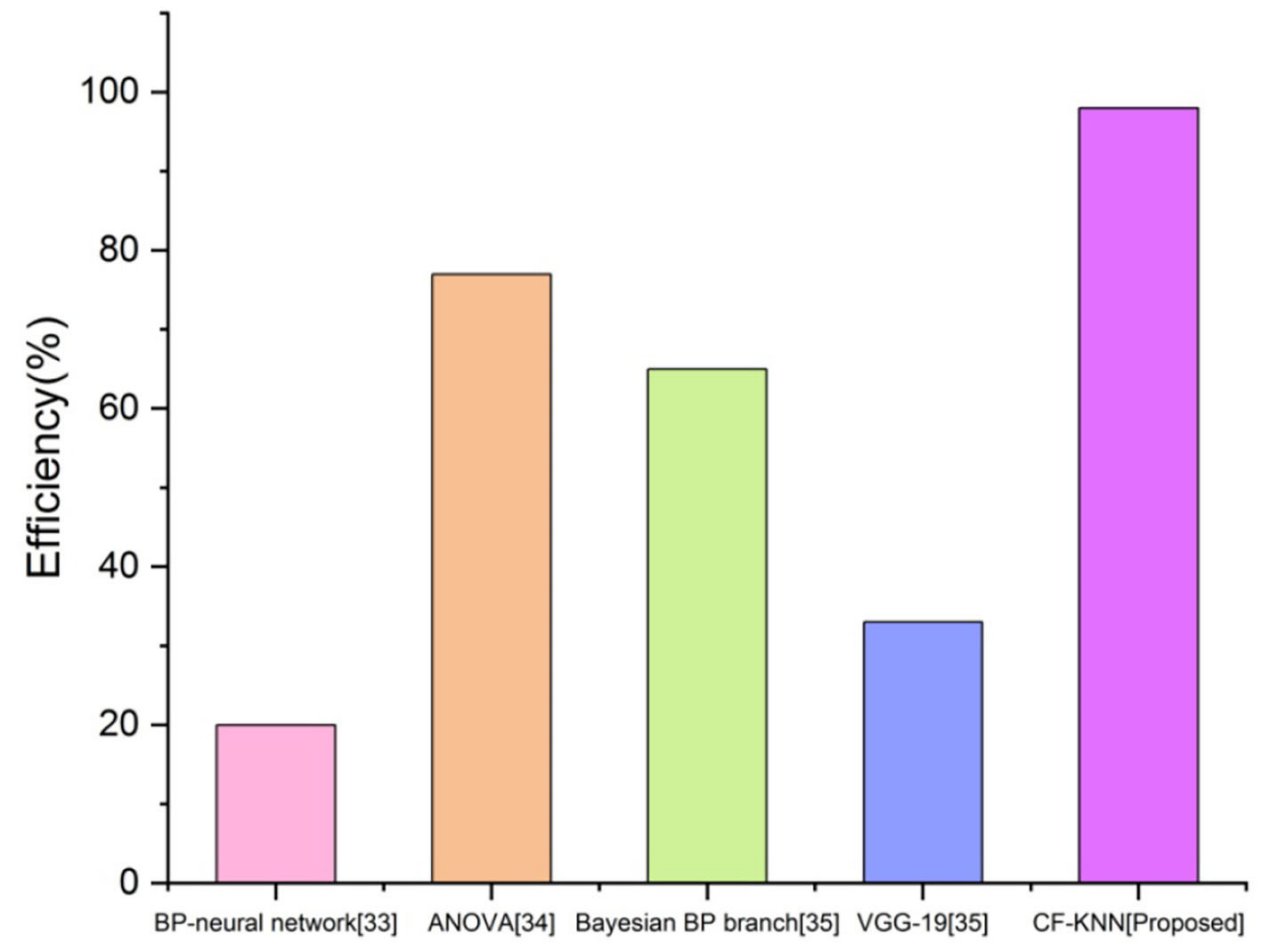
Comparison of efficiency with proposed and existing techniques.

A student's efficiency in AI is defined as being efficient as a condition or attribute. Figure 12 depicts the student efficiency of the planned technique for predicting students' mental health status by applying the AI model.

### Satisfaction

**Figure 13:**
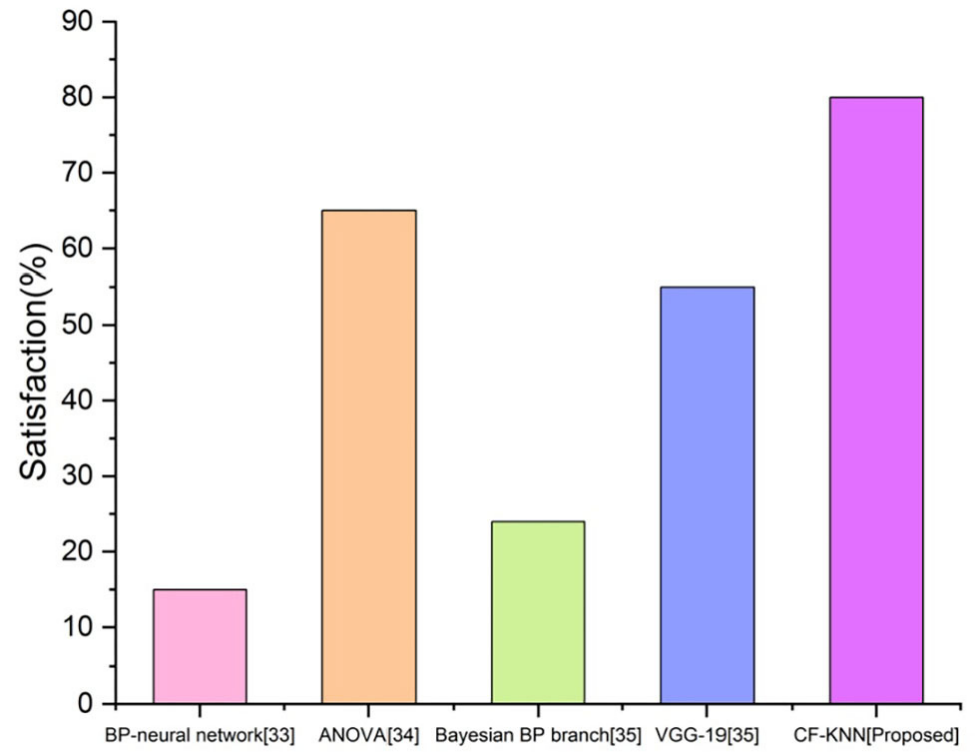
Comparison of satisfaction of proposed and existing techniques.

Student satisfaction in AI is described as a quick mindset arising from assessing the learning program, activities, and facilities provided to students. Figure 13 depicts the student's satisfaction with the proposed technique for predicting the student's mental health status by applying the AI model.

## Conclusion

The ability and legality of young adults may now be identified through law enforcers at this point. Understanding that institutions possess sensible legislation enables pupils to investigate health and life advancement properly. They got picked out legally owned guns to protect them and college kids against assaults and boost their sense of safety. Law students were able to learn and achieve together to improve their legal awareness, develop legal awareness, fight against regulations and violations, and execute the nation's efficient guide with law. Thus, the CF-KNN approach was presented to anticipate students' legal awareness and mental health status through AI usage. Firstly, the legal education student's datasets were carried out and pre-processed by employing the normalization approach and variational autoencoder (VAE). Correspondingly, multi-gradient ant colony optimization (MG-ACO) approaches were utilized to retrieve the features and select the feature sub-sets in feature extraction and feature selection stages. The exploration of our work is explained in performance analysis and specific performance metrics such as learning interest, learning accuracy, sensitivity, and specificity. Error rates are also assessed through calculations, indicating the efficiency of the proposed methodology for predicting student mental health status using AI. The proposed work is examined and compared with classical techniques to gain our work with the greatest effectiveness. Findings are depicted in chart formation by employing the MATLAB tool. T-C4.5 and F-LRA techniques were applied to assess adolescents' mental health conditions. Lastly, our suggested technique was allied to the other existing techniques to accomplish our work with supreme effectiveness through applying the AI model.

## Data Availability Statement

The raw data supporting the conclusions of this article will be made available by the authors, without undue reservation.

## Ethics Statement

Ethical review and approval was not required for the study on human participants in accordance with the local legislation and institutional requirements. The patients/participants provided their written informed consent to participate in this study.

## Author Contributions

The author confirms being the sole contributor of this work and has approved it for publication.

## Conflict of Interest

The author declares that the research was conducted in the absence of any commercial or financial relationships that could be construed as a potential conflict of interest.

## Publisher's Note

All claims expressed in this article are solely those of the authors and do not necessarily represent those of their affiliated organizations, or those of the publisher, the editors and the reviewers. Any product that may be evaluated in this article, or claim that may be made by its manufacturer, is not guaranteed or endorsed by the publisher.
